# Aptamer Embedded Arch-Cruciform DNA Assemblies on 2-D VS_2_ Scaffolds for Sensitive Detection of Breast Cancer Cells

**DOI:** 10.3390/bios11100378

**Published:** 2021-10-08

**Authors:** Jinfeng Quan, Yihan Wang, Jialei Zhang, Kejing Huang, Xuemei Wang, Hui Jiang

**Affiliations:** 1 State Key Laboratory of Bioelectronics, National Demonstration Center for Experimental Biomedical Engineering Education, School of Biological Science and Medical Engineering, Southeast University, Nanjing 210096, China; jfquan0224@163.com (J.Q.); yihanwangxynu@163.com (Y.W.); 220181811@seu.edu.cn (J.Z.); 2 School of Chemistry and Chemical Engineering, Guangxi University for Nationalities, Nanning 530008, China; kejinghuang@163.com

**Keywords:** MCF-7 cells, electrochemistry, 2-D materials, signal amplification, DNA assembly

## Abstract

Arch-cruciform DNA are self-assembled on AuNPs/VS_2_ scaffold as a highly sensitive and selective electrochemical biosensor for michigan cancer foundation-7 (MCF-7) breast cancer cells. In the construction, arch DNA is formed using two single-strand DNA sequences embedded with the aptamer for MCF-7 cells. In the absence of MCF-7 cells, a cruciform DNA labeled with three terminal biotin is bound to the top of arch DNA, which further combines with streptavidin-labeled horseradish peroxidase (HRP) to catalyze the hydroquinone-H_2_O_2_ reaction on the electrode surface. The presence of MCF-7 cells can release the cruciform DNA and reduce the amount of immobilized HRP, thus effectively inhibiting enzyme-mediated electrocatalysis. The electrochemical response of the sensor is negatively correlated with the concentration of MCF-7 cells, with a linear range of 10~1 × 10^5^ cells/mL, and a limit of detection as low as 5 cells/mL (S/N = 3). Through two-dimensional materials and enzyme-based dual signal amplification, this biosensor may pave new ways for the highly sensitive detection of tumor cells in real samples.

## 1. Introduction

Breast cancer is the fifth leading cause of death among all cancers, and it is also the most common non-skin cancer in women [1,2]. The breast cancer cell line can be classified based on the status of three important receptors, including estrogen receptor (ER), progesterone receptor (PR), and human epithelial receptor-2 (HER2) [3]. MCF-7 (ER+PR+HER2-), a typical breast cancer cell line, accounts for more than two-thirds of the cell lines used in related studies, along with T47-D and MDA-MB-231 cells, which have been widely used for breast cancer modelling. It is noteworthy that breast cancer cells can endanger the lives of patients through proliferation and metastasis over a short time. Early diagnosis and treatment can be helpful to better understand the patients’ condition, and make appropriate treatment plan according to the degree of disease, which is very important to improve the survival rate of breast cancer [4,5,6].

At present, there are several common breast cancer screening methods, including computed tomography (CT) [7,8], magnetic resonance imaging (MRI) [9,10], positron emission computed tomography (PET) [11,12] and flow cytometry [13]. However, these techniques are usually expensive and time-consuming, and can lead to false-positive or negative results due to limited resolution [14]. Therefore, it is necessary to develop alternative molecular biological methods with high sensitivity, high accuracy and low cost for breast cancer diagnosis. There are numerous methods to detect MCF-7, including electrochemistry [15], electrochemiluminescence [16], colorimetry [17] and photoelectrochemical methods [18]. Aptamers, which are single-stranded DNA or RNA analogues to antibodies [19], have been attracting a lot of interest due to their advantages over traditional recognition molecules. As the recognition probe for cancer cells, aptamers can specifically bind and recognize proteins on the surface of cancer cells [4,20,21]. Based on aptamers, highly sensitive and selective biosensors can be constructed for the detection of MCF-7 cancer cells.

For this purpose, a careful choice needs to be made with regard to biosensing elements with multiple amplification capacity. The first issue is to seek an efficient scaffold to support the physical or biochemical amplification. Recently, two-dimensional (2D) matrices have received tremendous interest [22,23,24]. Among them, vanadium disulfide (VS_2_) is the most common vanadium-based transition metal dichalcogenide [25]. VS_2_ nanosheets have excellent conductivity and have been well used in high-performance rechargeable metal ion batteries with abundant metal ion storage sites and low ion diffusion barriers [26,27,28], photoelectrochemical water splitting and solar cells [29,30]. More importantly, VS_2_ has a high specific surface area and superior mechanical properties, which is conducive to the construction of an effective biosensor platform. For example, Tian et al. [31] constructed an electrochemical aptasensor for kanamycin based on VS_2_/AuNPs nanocomposites and CoFe_2_O_4_ nanoenzyme as signal amplifiers. The recognition of kanamycin by aptamers results in the decrease in nanoenzyme accumulation and the increase in electrochemical signals by methylene blue. Actually, it may be more rational to design aptasensors depending on both VS_2_ nanosheets and the self-assembled complementary DNA sequences.

**Scheme 1:**
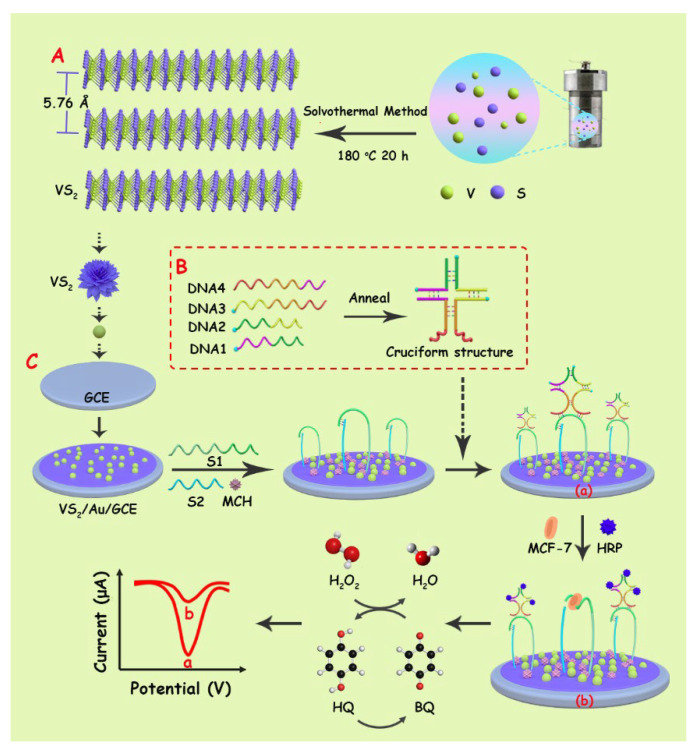
Illustration of highly sensitive breast cancer cell sensor based on VS_2_.

Hence, this work aims to construct a highly sensitive human breast MCF-7 cancer cell sensing platform with synergistic amplification by both VS_2_ nanocomposites and aptamer-binding enzyme (Scheme 1). Firstly, AuNPs are electrodeposited on the surfaces of VS_2_ nanosheets to fabricate an ideal electrochemical scaffold. Then, the arch DNA aptamer that specifically binds to MCF-7 cells is self-assembled on this scaffold through the well-known gold-thiolate interaction. In the absence of MCF-7 cells, the immobilized arch DNA is hybridized with biotin-labeled cruciform DNA and further linked with biotinylated horseradish peroxidase (HRP), which may catalyze the reaction of hydrogen peroxide (H_2_O_2_) and hydroquinone (HQ) to produce electrochemical signals. Target MCF-7 cells may compete with the cruciform DNA for the binding site of the arch DNA aptamer, and cause the release of the cruciform DNA, which further inhibits the immobilization of HRP on the electrode, thus reducing the electrochemical response. Based on this principle, the concentration of MCF-7 cells can be detected by the electrochemical signals. This biosensor may pave new ways for the highly sensitive detection of tumor cells in real samples.

## 2. Materials and Methods

### 2.1. Reagents and Instruments

Sodium orthovanadate (Na_3_VO_4_·12H_2_O), H_2_O_2_, 6-mercaptohexanol (MCH), horseradish peroxidase (HRP), and anhydrous ethanol were purchased from Sinopharm Chemical Reagent Co., Ltd. (Shanghai, China). Thioacetamide (CH_3_CSNH_2_), HQ, HAuCl_4_·4H_2_O, tris-(2-carboxyethyl) phosphine hydrochloride (TCEP) were purchased from Aladdin Co., Ltd. (Shanghai, China). All DNA strands used were purchased from Sangon Biotechnology Co., Ltd. (Shanghai, China), and the base sequences are listed in Table S1 (Supplementary Materials).

The details of instruments are listed in Table S2. The electrochemical experimental conditions and parameters are shown in Table S3.

### 2.2. Preparation of VS_2_ Nanosheets

VS_2_ nanosheets were prepared by the hydrothermal method. In a typical reaction, the precursors of sodium orthovanadate (2.36 g) and thioacetamide (2.79 g) were slowly dissolved in 60 mL of distilled water, and continuously stirred for 30 min to obtain a uniform solution. The solution was transferred to a 100 mL autoclave and kept at 180 °C for 20 h. After the reaction system was cooled to room temperature, the collected black precipitates were washed and centrifuged 3 times and dried at 60 °C for 12 h.

### 2.3. Preparation of Cruciform DNA

The cruciform DNA probe was assembled by four single strands of DNA, namely, DNA1, DNA2, DNA3 and DNA4. Firstly, the four single chains were mixed in the same molar ratio, and the final concentration of cruciform DNA was 1 μmol/L. Then, the mixture was heated to 95 °C for 10 min, and gradually cooled to 4 °C to obtain cruciform DNA probe by annealing steps (part B, Scheme 1). The successful preparation of cruciform DNA was verified by gel electrophoresis (Figure S2B). The cytotoxic assays also demonstrate the excellent biosafety of cruciform DNA (Figure S2A).

### 2.4. Cytotoxicity Test of Arch and Cruciform DNA

MCF-7 cells were seeded in 96-well plates at 1 × 10^4^/well. After 24 h, the cells completely adhered to the well and the medium was removed. After 24 h incubation with different concentrations of arch DNA and cruciform DNA, 10 μL CCK-8 solution was added in each well and incubated at 37 °C for 2 h in dark. The optical density of each well at 450 nm was measured by microplate, and the cell survival rate was calculated [32].

### 2.5. Preparation of Biosensor

Firstly, glassy carbon electrode (GCE) was pretreated with aluminum oxide powder, cleaned and dried. VS_2_ suspension (1 mg/mL) was dripped on the electrode surface and dried in air to obtain VS_2_/GCE. Then, VS_2_/GCE was immersed in a mixed solution containing 0.1% HAuCl_4_ and 0.1 mol/L KCl, and AuNPs were electrodeposited by the amperometric I-t method with deposition voltage of −0.2 V and deposition time of 25 s [33]. AuNPs were in situ synthesized on the electrode surface to form AuNPs/VS_2_/GCE. The mixed aptamer solution was obtained by dissolving 1 μmol/L aptamer S1 and 1 μmol/L aptamer S2 in Tris-HCl buffer of pH 7.0 (containing 50 mmol/L NaCl, 10 mmol/L MgCl_2_, and 10 mmol/L TCEP). An amount of 10 μL of the above mixed solution was coated on AuNPs/VS_2_/GCE and incubated at room temperature for 12 h. Both DNA strands were immobilized on the electrode by Au-S bond and electrostatic interaction. Note that S1 and S2 have complementary tail sequences to form arched DNA. After washing with distilled water, MCH (5 μL, 1 mmol/L) was incubated on the electrode surface for 30 min to block the active sites and inhibit the non-specific adsorption. An amount of 8 μL cruciform DNA probe was then modified on the electrode surface by incubation at 37 °C for 80 min, in virtue of the hybridization of cruciform DNA to the top of arch DNA. At each modification step, the electrode was washed with Tris-HCl buffer (pH = 7.4) to remove the unbound molecules.

For cellular measurements, different concentrations of cell suspension of 8 μL were incubated at room temperature for 100 min, and the aptamer S1 was used to capture the cells. Subsequently, 8 μL HRP (10 μg/mL) labeled with streptavidin was immobilized on the electrode surface at 37 °C for 30 min and bound to the cruciform DNA surface by biotin–streptavidin interaction. For DPV tests, the electrodes were placed in 10 mL 0.1 mol/L PBS (pH 5.0) containing 1.8 mmol/L H_2_O_2_ and 2 mmol/L HQ, respectively.

## 3. Results and Discussion

### 3.1. Characterization of VS_2_

**Figure 1:**
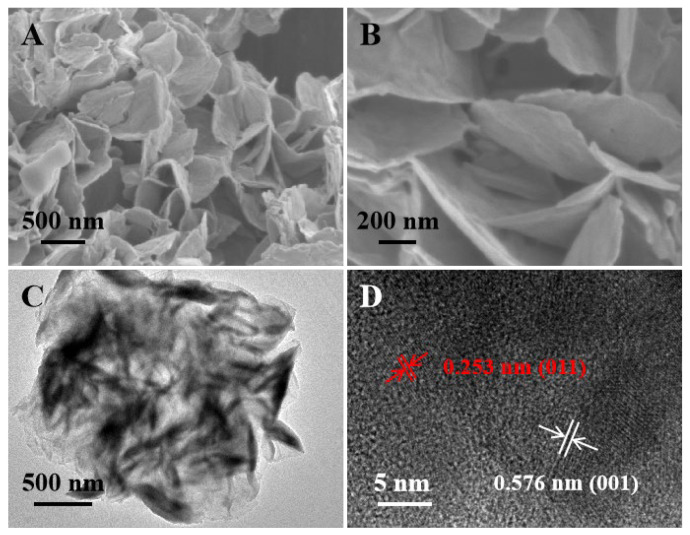
Morphological characterization of VS_2_ nanosheets: (**A**,**B**) SEM images; (**C**)TEM image; (**D**) HR-TEM image.

The morphology of VS_2_ is characterized in Figure 1. The SEM images show that a large number of 2D VS_2_ nanosheets are stacked and interwoven to form a flower-like structure (Figure 1A,B). These nanosheets are uniform in thickness (~30 nm) and the diameter ranges from 0.5 to 1 µm. The TEM image in Figure 1C show that single flower-like VS_2_ is composed of loosely arranged nanosheets, which are connected by the center to form three-dimensional layers. The thin layer extends outwards and provides an excellent electron transfer interface. Figure 1D shows a high-resolution transmission electron microscope (HR-TEM) image inside a nanoplate, showing that the interlayer spacing is about 0.576 and 0.253 nm, corresponding to the (001) and (011) crystal plane of VS_2_ [34], respectively.

**Figure 2:**
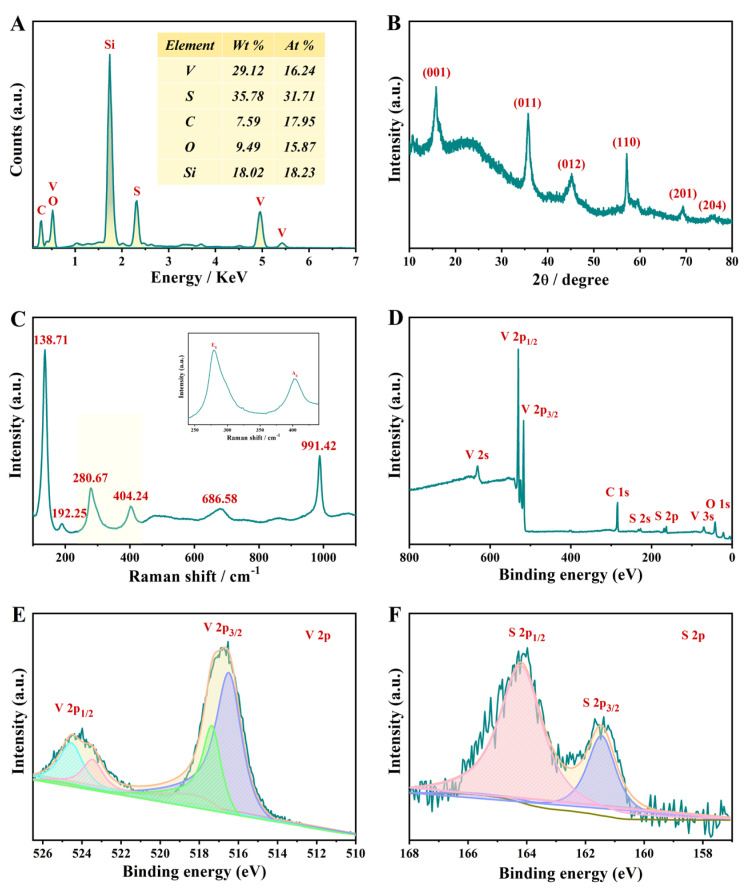
Characterization of VS_2_ nanosheets: (**A**) EDS; (**B**) XRD; (**C**) Raman; (**D**) XPS. (**E**,**F**) show the amplified V 2p and S 2p zone in XPS.

The distribution of elemental components in VS_2_ was studied by energy-dispersive X-ray spectroscopy (EDS) (Figure 2A). The results show the presence of V, S, C, and O on the surface of substrate. The atomic number ratio of V to S is about 1:1.95, which is very close to the theoretical atomic number ratio of 1:2 of VS_2_, indicating a high purity. The XRD results (Figure 2B) show that the diffraction peaks of VS_2_ nanosheets are located at 15.41°, 36.12°, 45.46°, 57.15°, 69.23° and 75.25°, which can be attributed to the corresponding diffraction crystal planes of (001), (011), (012), (110), (201) and (202), respectively, for the hexagonal phase of 2H-VS_2_ (JPCDS No. 89-1640). Moreover, the sharp (011) diffraction peak proves that VS_2_ has good crystallinity and layered structure [27]. In Raman spectroscopy, the characteristic peaks of VS_2_ are 138.71 cm^−1^, 192.25 cm^−1^, 282.67 cm^−1^, 404.24 cm^−1^, 686.58 cm^−1^ and 991.42 cm^−1^ in the range of 100~1100 cm^−1^ (Figure 2C). Typically, the peaks at 138.71 cm^−1^ and 192.25 cm^−1^ can be attributed to the vibration dispersion of VS_2_. The characteristic peak at 282.67 cm^−1^ corresponds to the plane (E_1g_) vibration mode in VS_2_, which is the opposite vibration of two S atoms relative to V atoms in the plane; the peak at 404.24 cm^−1^ corresponds to the out of plane (A_1g_) vibration mode of the S atom along the c axis [34]. The small half peak width and high intensity of these peaks also indicate that the VS_2_ samples are in a highly crystalline state. The XPS (Figure 2D) also shows the V, S, C and O elements in samples, which is consistent with the EDS results. The two binding energy peaks of V 2p spectrum (Figure 2E) appear at 524.3 eV and 516.7 eV, which could correspond to the molecular orbits of V 2p_1/2_ and V 2p_3/2_, indicating that V exists at + 4 valence in VS_2_. The S 2p_3/2_ and S 2p_1/2_ peaks are located at 161.4 and 164.24 eV, respectively (Figure 2F), proving that the presence of −2 valence [30].

### 3.2. Electrochemical Characterization

To fabricate the biosensor, we used a dual amplification strategy on the basis of AuNPs/VS_2_ nanocomposites and enzyme, using aptamers with highly addressable and strong affinity, selectivity and stability. Considering that 2D and 3D DNA structures have stronger selective binding affinity and better stability compared to other common nucleic acid structures, an arch-cruciform-shaped DNA is introduced, which can combine with more enzymes for signal amplification.

In principle, when the cell target does not exist, cruciform DNA with biotin at three ends will hybridize with the sequence on the top of the arched DNA surface. Then, HRP labeled with streptavidin is immobilized by the streptavidin–biotin interaction, which can further catalyze the reaction of HQ and H_2_O_2_ on the electrode surface. In the presence of tumor cells, the cruciform DNA is released due to the affinity between the aptamer and the target, which effectively inhibits the sequent immobilization of HRP on the electrode surface, resulting in a weak electrical signal. Therefore, the electrochemical signal is negatively correlated with the concentration of the target (Scheme 1). In this strategy, AuNPs/VS_2_ plays a crucial role of signal amplification (Figure S1). Moreover, it may also provide a solid scaffold for the immobilization of arch DNA and cruciform DNA, and the latter further provides a large number of HRP loading sites, enabling the enzyme-mediated signal amplification. Therefore, a dual signal amplification system was fabricated by this design.

**Figure 3:**
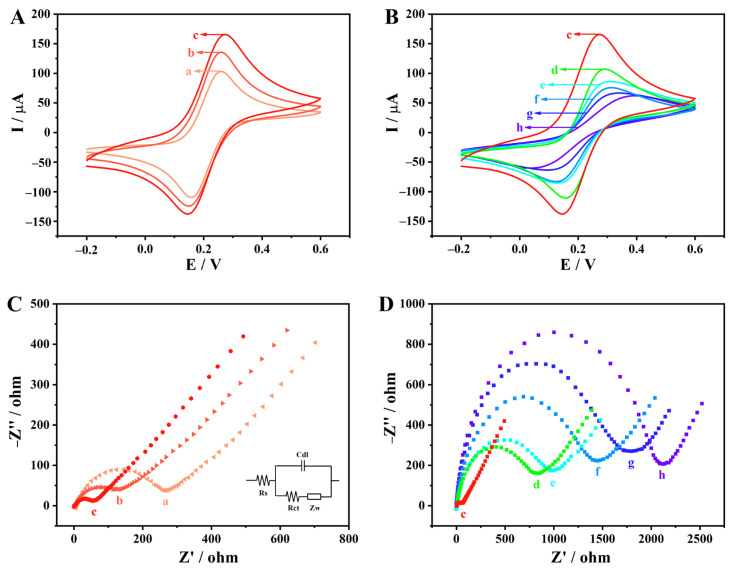
CV (**A**,**B**) and EIS (**C**,**D**) of different electrodes: (a) GCE; (b) VS_2_/GCE; (c) AuNPs/VS_2_/GCE; (d) arch DNA/AuNPs/VS_2_/GCE; (e) MCH/arch DNA/AuNPs/VS_2_/GCE; (f) cruciform DNA/MCH/arch DNA/AuNPs/VS_2_/GCE; (g) target cell/cruciform DNA/MCH/arch DNA/AuNPs/VS_2_/GCE; (h) HRP/target cell/cruciform DNA/MCH/arch DNA/AuNPs/VS_2_/GCE. The inner illustration of (C) is the corresponding equivalent circuit diagram of the reaction system. Rct, Rs, Zw and Cdl in the circuit represent charge transfer resistance, solution resistance, Warburg impedance and electric double layer capacitance, respectively. Electrolyte: 0.1 M KCl containing 5 mM [Fe(CN)_6_]^3-/4-^.

The electrochemical characteristics on a series of modified electrodes were investigated by CV (Figure 3A,B) and EIS (Figure 3C,D). Firstly, the modification of VS_2_ increases the electron transfer rate and the surface roughness of the electrode, resulting in a 1.3-fold increase in the electrochemical response (curve b) compared with bare GCE (curve a). Furthermore, the redox currents further increase by 27% upon the deposition of AuNPs on the modified electrode, giving AuNPs/VS_2_/GCE (curve c). When the arch DNA is immobilized on the electrode surface (curve d), the negatively charged DNA strand hinders the electron transport of ferricyanide, and the redox currents decrease to a level similar to those on the bare electrode (curve a). After the treatment of blocking agent MCH (curve e), the non-specific binding sites on the electrode surface are blocked, and the electrochemical signal further decreases by 15%. With the modification of cruciform DNA (curve f) and target MCF-7 cells (curve g), the electrostatic repulsion and steric hindrance induced by the DNA strand and cells further passivate the electrode surface. The last step of modification is to immobilize HRP to the biotin-labeled cruciform DNA strand, and it shows the lowest electrochemical signal due to the low conductivity of proteins (curve h).

Correspondingly, in the EIS plot (Figure 3C,D), the *R_ct_* value of GCE is 260 Ω for bare GCE (curve a). After the electrodes are modified with VS_2_ (curve b) and AuNPs/VS_2_ (curve c), the *R_ct_* value continues to decrease to 132 Ω and 67 Ω, respectively, proving the good conductivity of nanostructures. When arch DNA, MCH and cruciform DNA are added to the electrode surface in turn (curves d, e and f), the *R_ct_* value increases greatly to 810 Ω, 960 Ω, and 1432 Ω, respectively, since both negatively charged DNA strands and MCH can reduce the electron transfer efficiency and increase the resistance of the system [35]. Finally, with the further immobilization of target cell MCF-7 and biotin-tagged HRP on the electrode surface (curve g and h), the *R_ct_* value increases to 1710 Ω and 2053 Ω. The analysis results of EIS and CV are consistent, which proves the successful preparation of the sensor. 

### 3.3. Optimization of Experimental Conditions

**Figure 4:**
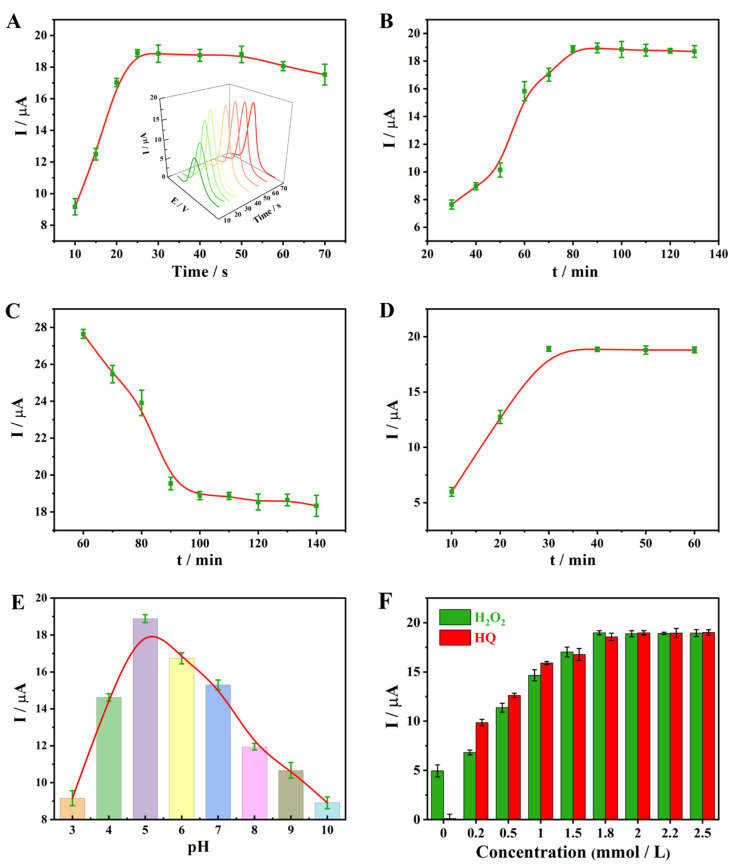
Optimization of experimental conditions: (**A**) deposition time of AuNPs; (**B**) incubation time of cruciform DNA; (**C**) incubation time of cells; (**D**) incubation time of HRP; (**E**) pH optimization; (**F**) concentration of H_2_O_2_ and HQ.

Under a cell concentration of 5000 cells/mL, the experimental conditions were optimized. Figure 4A shows the relationship of the DPV current and electrodeposition time for AuNPs. During the period of 10~25 s, the electrochemical response increases continuously, which corresponds to the stronger conductivity caused by AuNPs immobilized on the electrode. In the period of 25~70 s, the AuNP particles tend to be saturated, and the peak current basically remains stable. Therefore, 25 s is the best time for the electrodeposition of AuNPs.

In addition to the deposition of Au NPs, the immobilization of the DNA strand is time-dependent. Figure 4B shows the DPV response after incubation cruciform DNA with a different time. When the incubation time is less than 80 min, the current continues to rise, indicating that the longer the incubation time is, the more HRP is bound. However, after 80 min, the current reaches stability and does not increase thereafter, due to the saturation of HRP loaded by cruciform DNA. The best incubation time for cruciform DNA is 80 min.

In addition, the cell incubation time is also an important parameter. As shown in Figure 4C, the cells gradually replace the cruciform DNA on arch DNA within 60~100 min, causing a reduction in the amount of immobilized cruciform DNA attached with HRP, as well as the enzyme catalyzed electrochemical signals. When the incubation time is more than 100 min, the peak current of DPV remains stable and does not decrease significantly, indicating that the cell incubation reaches equilibrium. 

The incubation of biotin-labeled HRP can affect the performance of the sensor (Figure 4D). When the incubation time is less than 30 min, the immobilized amount of HRP increases with time, and the peak current increases gradually. After 30 min, the immobilized HRP on the electrode surface tends to be saturated. Therefore, the best time for HRP immobilization is 30 min. Since the pH and substrate concentrations are vital for HRP catalyzed reaction, this system was further optimized, obtaining an optimal pH of 5 (Figure 4E) and an optimal substrate concentration of 1.8 mmol/L (H_2_O_2_) and 2.0 mmol/L (HQ), respectively (Figure 4F).

### 3.4. Analysis Performance of Sensor

**Figure 5:**
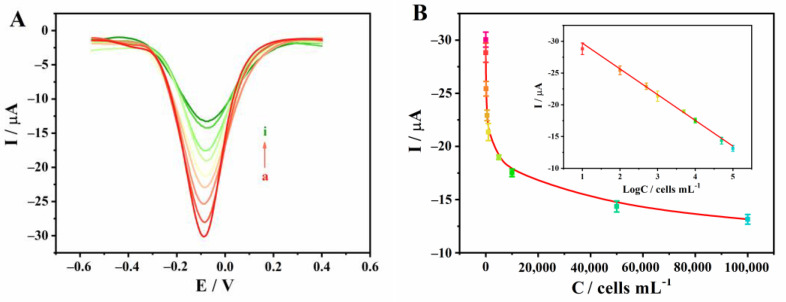
Detection performance of the sensor: (**A**) DPV curves of different concentrations of MCF-7, with the concentration of a~i: 0, 10, 10^2^, 5 × 10^2^, 1 × 10^3^, 5 × 10^3^, 1 × 10^4^, 5 × 10^4^, 1 × 10^5^ cells/mL, respectively; (**B**) the relationship between the DPV current and the concentration of MCF-7. The inner illustration shows the linear relationship between the peak current of DPV and the logarithm of the MCF-7 cell concentration. The error bar represents the standard deviation of three measurements.

Under the above optimal experimental conditions, the sensor was used to detect different concentrations of MCF-7 cells. As shown in Figure 5A, the DPV signal gradually decreases with the increasing concentration of target cells. The DPV response is linearly correlated with the logarithm of MCF-7 cell concentration (Figure 5B, and 5B inset). The linear equation is I (μA) = 4.15 × log c (cells/mL) − 33.74, with a correlation coefficient R of 0.993. The linear range is 10 ~ 1 × 10^5^ cells/mL, and the detection limit is 5 cells/mL (S/N = 3). In comparison, the linear range and limit of detection (LOD) for the reported MCF-7 sensors are listed in Table 1. The as-prepared MCF-7 sensor has a low LOD and a wide linear range among the cases, showing excellent electrochemical performances.

### 3.5. Specificity, Reproducibility, Stability and Real Sample Analysis

**Figure 6:**
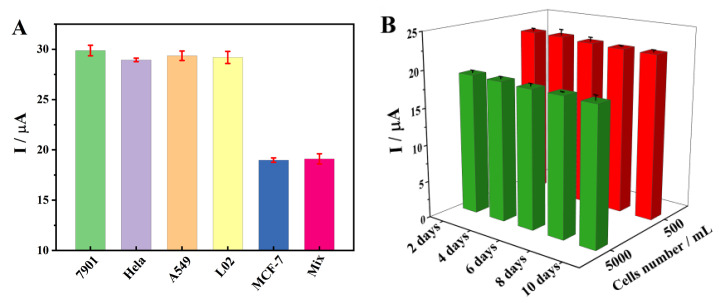
The specificity (**A**) and stability (**B**) of MCF-7 cell sensor.

The specificity of the sensor was investigated. In Figure 6A, the DPV responses to SGC-7901, HeLa, A549, L02 and MCF-7 cell suspensions with a concentration of 5000 cells/mL were tested, respectively. Only MCF-7 cells can significantly reduce the DPV signal, while in the presence of the other cells they exhibit a large electrochemical response, i.e., negligible cell attachment to the electrode interface. There is no significant difference in the current between a mixture of MCF-7 and L02 cell suspensions with the same concentrations and that of a pure MCF-7 cell suspension, which further proves that the prepared sensor has good specificity for MCF-7 cells.

Next, the reproducibility of the sensor is explored. Six parallel sensors were prepared to detect MCF-7 cells with a concentration of 500 cells/mL. The RSD of DPV peak current is 4.5%, indicating that the sensor has good reproducibility. In order to verify the storage stability, 10 electrodes were prepared in parallel and stored in a refrigerator at 4 °C for 10 days. According to the concentration of MCF-7 cells, they were divided into two groups: 500 cells/mL and 5000 cells/mL, with five electrodes in each group. The change of the DPV current was detected every two days (Figure 6B). There is no significant difference in the peak current between the two groups, indicating that the sensor has good stability.

In order to verify the application of the sensor in real samples, the recovery rates of MCF-7 in PBS and 10 times diluted whole-blood samples were tested, as shown in Table 2. For the concentration range of MCF-7 cells from 10 to 5 × 10^3^ cells/mL, the recovery rates of MCF-7 spiked in PBS and diluted blood samples are 90.0~103.2% and 77.8~84.1%, respectively. The main reason for the low recovery rate in blood samples may be caused by many potential interferences such as red blood cells, white blood cells and high concentrated proteins in whole blood.

## 4. Conclusions

We have proposed a dual amplification strategy for the fabrication of a highly sensitive electrochemical biosensor for MCF-7 cells. In principle, AuNPs/VS_2_ composites cannot only efficiently amplify the electrochemical signals due to their high conductivity, but also provide a solid scaffold for the hybridization of arch DNA and biotin-terminated cruciform DNA, as well as the following attachment of streptavidin-labeled HRP, which allows further enzyme catalyzed amplification. MCF-7 cells may competitively bind to the arch DNA and cause the release of cruciform DNA, which decreases the final amount of HRP immobilized on the electrode surface, thus reducing the electrochemical response by HRP catalysis. The DPV current is negatively correlated with the concentration of the target cells, with a linear range of 10 ~ 1 × 10^5^ cells/mL, and a LOD of 5 cells/mL. In addition to good anti-interference ability to other types of cells, the biosensor may be applied for the detection of spiked cells in blood samples, suggesting its great applicability in the early diagnosis of breast cancer.

## Figures and Tables

**Table 1:** Comparison between the constructed sensor and other sensors for detecting cells.

Detection Techniques	Experimental Methods	Linear Range (cell/mL)	LOD (cell/mL)	Ref.
EIS	Zr-MOF compound material	1 × 10^2^ ~ 1 × 10^5^	31	[36]
ICP-MS	Aptamer based bifunctional probe	2 × 10^2^ ~ 1.2 × 10^4^	81	[37]
Colorimetry	PH-AuPd-NPs combined 3D-rGO	50 ~ 1 × 10^7^	32	[38]
DPV	AuNGs/MWCNT-NH_2_	1 × 10^2^ ~ 1 × 10^6^	80	[39]
CC	DNA walker	0 ~ 5 × 10^2^	47	[40]
ICP-MS	Magnetic bead binding anti-EpCAM	2 × 10^2^ ~ 4 × 10^3^	50	[41]
EIS	Clay-protein based nanocomposites	1.5 × 10^2^ ~ 7.5 × 10^6^	148	[42]
DPV	Branched chain peptide modified electrode interface	50 ~ 1 × 10^6^	20	[43]
Fluorescence	aptamer-modified magnetic beads	10 ~ 1 × 10^5^	5	[44]
DPV	BSA@Ag@Ir metallic-organic nanoclusters	3 ~ 3 × 10^6^	1	[45]
DPV	Arch DNA, cruciform DNA, material signal amplification, enzyme amplification	10 ~ 1 × 10^5^	5	This work

ICP-MS: inductively coupled plasma mass spectrometry.

**Table 2:** Detection of MCF-7 in PBS and human whole-blood samples (n = 3).

Sample	Concentration (cells/mL)		Recovery Rate (%)
	Amount Added	Measured Value	
PBS	10	9	90.0
	100	92	92.0
	500	479	95.8
	1000	983	98.3
	2000	2063	103.2
	5000	4760	95.2
Blood sample	10	8	80.0
	100	83	83.0
	500	389	77.8
	1000	841	84.1

## Data Availability

The data presented in this study are available in supplementary material.

## Supplementary Materials

The following are available online at https://www.mdpi.com/article/10.3390/bios11100378/s1, Figure S1: Signal amplification of nanocomposites: (A) Q-t curves of GCE and AuNPs/VS2/GCE, with Q-t1/2 curves of GCE and AuNPs/VS2/GCE in the inner illustration; (B) The DPV responses of HRP/target cell/cruciform DNA/MCH/arch-DNA structure/AuNPs/GCE (a) and HRP/target cell/cruciform DNA/MCH/arch-DNA structure/AuNPs/VS2/GCE (b). Figure S2: (A) Cytotoxicity test of arch DNA (green) and cruciform DNA (red); (B) Cruciform DNA electrophoresis. Lane 1–9: DNA1, DNA2, DNA3, DNA4, DNA1+DNA2, DNA3+DNA4, DNA1+DNA2+DNA3, DNA2+DNA3+DNA4, DNA1+DNA2+DNA3+DNA4. Table S1: DNA sequences. Table S2: List of instruments. Table S3: Experimental conditions and electrochemical parameters.

## References

[1] Wang K., He M.-Q., Zhai F.-H., He R.-H., Yu Y.-L. (2017). A novel electrochemical biosensor based on polyadenine modified aptamer for label-free and ultrasensitive detection of human breast cancer cells. Talanta.

[2] Li T., Fan Q., Liu T., Zhu X., Zhao J., Li G. (2010). Detection of breast cancer cells specially and accurately by an electrochemical method. Biosens. Bioelectron..

[3] Dai X., Cheng H., Bai Z., Li J. (2017). Breast Cancer Cell Line Classification and Its Relevance with Breast Tumor Subtyping. J. Cancer.

[4] Sheng Q., Cheng N., Bai W., Zheng J. (2015). Ultrasensitive electrochemical detection of breast cancer cells based on DNA-rolling-circle-amplification-directed enzyme-catalyzed polymerization. Chem. Commun..

[5] Liu R., Wang Q., Li Q., Yang X., Wang K., Nie W. (2017). Surface plasmon resonance biosensor for sensitive detection of microRNA and cancer cell using multiple signal amplification strategy. Biosens. Bioelectron..

[6] Li Y., Huan K., Deng D., Tang L., Wang J., Luo L. (2019). Facile Synthesis of ZnMn2O4@rGO Microspheres for Ultrasensitive Electrochemical Detection of Hydrogen Peroxide from Human Breast Cancer Cells. ACS Appl. Mater. Interfaces.

[7] Bernsdorf M., Berthelsen A.K., Wielenga V.T., Kroman N., Teilum D., Binderup T., Tange U.B., Andersson M., Kjær A., Loft A. (2012). Preoperative PET/CT in early-stage breast cancer. Ann. Oncol..

[8] Luo Y., Pan Q., Yang H., Peng L., Zhang W., Li F. (2021). Fibroblast Activation Protein–Targeted PET/CT with 68Ga-FAPI for Imaging IgG4-Related Disease: Comparison to 18F-FDG PET/CT. J. Nucl. Med..

[9] Warner E., Plewes D.B., Hill K.A., Causer P.A., Zubovits J.T., Jong R.A., Cutrara M.R., DeBoer G., Yaffe M.J., Messner S.J. (2004). Surveillance of BRCA1 and BRCA2 Mutation Carriers With Magnetic Resonance Imaging, Ultrasound, Mammography, and Clinical Breast Examination. JAMA.

[10] Hananouchi T., Chen Y., Jerban S., Teramoto M., Ma Y., Dorthe E., Chang E., Du J., D’Lima D. (2021). A Useful Combination of Quantitative Ultrashort Echo Time MR Imaging and a Probing Device for Biomechanical Evaluation of Articular Cartilage. Biosensors.

[11] Galgano S., Viets Z., Fowler K., Gore L., Thomas J.V., McNamara M., McConathy J. (2017). Practical Considerations for Clinical PET/MR Imaging. Magn. Reson. Imaging Clin. N. Am..

[12] Giesel F.L., Adeberg S., Syed M., Lindner T., Jiménez-Franco L.D., Mavriopoulou E., Staudinger F., Tonndorf-Martini E., Regnery S., Rieken S. (2021). FAPI-74 PET/CT Using Either 18F-AlF or Cold-Kit 68Ga Labeling: Biodistribution, Radiation Dosimetry, and Tumor Delineation in Lung Cancer Patients. J. Nucl. Med..

[13] Galanzha E.I., Kim J.-W., Zharov V.P. (2009). Nanotechnology-based molecular photoacoustic and photothermal flow cytometry platform forin-vivodetection and killing of circulating cancer stem cells. J. Biophotonics.

[14] Mittal S., Kaur H., Gautam N., Mantha A.K. (2017). Biosensors for breast cancer diagnosis: A review of bioreceptors, biotransducers and signal amplification strategies. Biosens. Bioelectron..

[15] Wang Q., Zou L., Yang X., Liu X., Nie W., Zheng Y., Cheng Q., Wang K. (2019). Direct quantification of cancerous exosomes via surface plasmon resonance with dual gold nanoparticle-assisted signal amplification. Biosens. Bioelectron..

[16] Su M., Liu H., Ge L., Wang Y., Ge S., Yu J., Yan M. (2014). Aptamer-Based electrochemiluminescent detection of MCF-7 cancer cells based on carbon quantum dots coated mesoporous silica nanoparticles. Electrochim. Acta.

[17] Wang X., Cheng S., Wang X., Wei L., Kong Q., Ye M., Luo X., Xu J., Zhang C., Xian Y. (2021). pH-Sensitive Dye-Based Nanobioplatform for Colorimetric Detection of Heterogeneous Circulating Tumor Cells. ACS Sens..

[18] Luo J., Liang D., Li X., Deng L., Wang Z., Yang M. (2020). Aptamer-based photoelectrochemical assay for the determination of MCF-7. Microchim. Acta.

[19] Huang R., Xi Z., He N. (2015). Applications of aptamers for chemistry analysis, medicine and food security. Sci. China Ser. B Chem..

[20] Vajhadin F., Ahadian S., Travas-Sejdic J., Lee J., Mazloum-Ardakani M., Salvador J., Aninwene G.E., Bandaru P., Sun W., Khademhossieni A. (2020). Electrochemical cytosensors for detection of breast cancer cells. Biosens. Bioelectron..

[21] Lu F., Yang L., Hou T., Li F. (2020). Label-free and “signal-on” homogeneous photoelectrochemical cytosensing strategy for ultrasensitive cancer cell detection. Chem. Commun..

[22] Zhu D., Liu B., Wei G. (2021). Two-Dimensional Material-Based Colorimetric Biosensors: A Review. Biosensors.

[23] Kasani S.P.K., Curtin K., Wu N. (2019). A review of 2D and 3D plasmonic nanostructure array patterns: Fabrication, light management and sensing applications. Nanophotonics.

[24] Wang Y.-H., Huang K.-J., Wu X. (2017). Recent advances in transition-metal dichalcogenides based electrochemical biosensors: A review. Biosens. Bioelectron..

[25] Yu D., Pang Q., Gao Y., Wei Y., Wang C., Chen G., Du F. (2018). Hierarchical flower-like VS2 nanosheets — A high rate-capacity and stable anode material for sodium-ion battery. Energy Storage Mater..

[26] Li W., Sari H.M.K., Li X. (2020). Emerging Layered Metallic Vanadium Disulfide for Rechargeable Metal-Ion Batteries: Progress and Opportunities. ChemSusChem.

[27] Xua D., Wanga H., Qiua R., Wangb Q., Maoa Z., Jiangc Y., Wanga R., Hea B., Gonga Y., Lib D. (2020). Coupling of bowl-like VS2 nanosheet arrays and carbon nanofiber enables ultrafast Na+-Storage and robust flexibility for sodium-ion hybrid capacitors. Energy Storage Mater..

[28] Mikhaleva N.S., Visotin M.A., Kuzubov A.A., Popov Z.I. (2017). VS2/Graphene Heterostructures as Promising Anode Material for Li-Ion Batteries. J. Phys. Chem. C.

[29] Liu Y.-Y., Xu L., Guo X.-T., Lv T.-T., Pang H. (2020). Vanadium sulfide based materials: Synthesis, energy storage and conversion. J. Mater. Chem. A.

[30] Xie X.-C., Shuai H.-L., Wu X., Huang K.-J., Wang L.-N., Wang R.-M., Chen Y. (2020). Engineering ultra-enlarged interlayer carbon-containing vanadium disulfide composite for high-performance sodium and potassium ion storage. J. Alloy. Compd..

[31] Tian L., Zhang Y., Wang L., Geng Q., Liu D., Duan L., Wang Y., Cui J. (2020). Ratiometric Dual Signal-Enhancing-Based Electrochemical Biosensor for Ultrasensitive Kanamycin Detection. ACS Appl. Mater. Interfaces.

[32] Wang M., Chen Y., Cai W., Feng H., Du T., Liu W., Jiang H., Pasquarelli A., Weizmann Y., Wang X. (2020). In situ self-assembling Au-DNA complexes for targeted cancer bioimaging and inhibition. Proc. Natl. Acad. Sci. USA.

[33] Chen Y.-X., Huang K.-J., Lin F., Fang L.-X. (2017). Ultrasensitive electrochemical sensing platform based on graphene wrapping SnO2 nanocorals and autonomous cascade DNA duplication strategy. Talanta.

[34] He P., Yan M., Zhang G., Sun R., Chen L., An Q., Mai L. (2017). Layered VS2 Nanosheet-Based Aqueous Zn Ion Battery Cathode. Adv. Energy Mater..

[35] Huang L., Deng H., Zhong X., Zhu M., Chai Y., Yuan R., Yuan Y. (2019). Wavelength distinguishable signal quenching and enhancing toward photoactive material 3,4,9,10-perylenetetracarboxylic dianhydride for simultaneous assay of dual metal ions. Biosens. Bioelectron..

[36] Li Y., Hu M., Huang X., Wang M., He L., Song Y., Jia Q., Zhou N., Zhang Z., Du M. (2020). Multicomponent zirconium-based metal-organic frameworks for impedimetric aptasensing of living cancer cells. Sens. Actuators B Chem..

[37] Yang B., Chen B., He M., Yin X., Xu C., Hu B. (2018). Aptamer-Based Dual-Functional Probe for Rapid and Specific Counting and Imaging of MCF-7 Cells. Anal. Chem..

[38] Wang H., Zhou C., Sun X., Jian Y., Kong Q., Cui K., Ge S., Yu J. (2018). Polyhedral-AuPd nanoparticles-based dual-mode cytosensor with turn on enable signal for highly sensitive cell evalution on lab-on-paper device. Biosens. Bioelectron..

[39] Yang Y., Fu Y., Su H., Mao L., Chen M. (2018). Sensitive detection of MCF-7 human breast cancer cells by using a novel DNA-labeled sandwich electrochemical biosensor. Biosens. Bioelectron..

[40] Cai S., Chen M., Liu M., He W., Liu Z., Wu D., Xia Y., Yang H., Chen J. (2016). A signal amplification electrochemical aptasensor for the detection of breast cancer cell via free-running DNA walker. Biosens. Bioelectron..

[41] Li X., Chen B., He M., Wang H., Xiao G., Yang B., Hu B. (2017). Simultaneous detection of MCF-7 and HepG2 cells in blood by ICP-MS with gold nanoparticles and quantum dots as elemental tags. Biosens. Bioelectron..

[42] Yaman Y.T., Akbal Ö., Abaci S. (2019). Development of clay-protein based composite nanoparticles modified single-used sensor platform for electrochemical cytosensing application. Biosens. Bioelectron..

[43] Liu N., Song J., Lu Y., Davis J.J., Gao F., Luo X. (2019). Electrochemical Aptasensor for Ultralow Fouling Cancer Cell Quantification in Complex Biological Media Based on Designed Branched Peptides. Anal. Chem..

[44] Shenab C., Zhongb L., Xiongab L., Liub C., Yuc L., Chuab X., Luoab X., Zhaob M., Liuab B. (2021). A novel sandwich-like cytosensor based on aptamers-modified magnetic beads and carbon dots/cobalt oxyhydroxide nanosheets for circulating tumor cells detection. Sens. Actuators B Chem..

[45] Shen H., Liu L., Yuan Z., Liu Q., Li B., Zhang M., Tang H., Zhang J., Zhao S. (2021). Novel cytosensor for accurate detection of circulating tumor cells based on a dual-recognition strategy and BSA@Ag@Ir metallic-organic nanoclusters. Biosens. Bioelectron..

