# Supplementary material for: Aptamer Embedded Arch-Cruciform DNA Assemblies on 2-D VS2 Scaffolds for Sensitive Detection of Breast Cancer Cells
Source: Biosensors (Basel). 2021 Oct 8;11(10):378. doi: 10.3390/bios11100378 (PMC8534259; doi:10.3390/bios11100378)
Supplement: Supplementary file 1 [file biosensors-11-00378-s001.zip › biosensors-1388702-supplementary.pdf]

Supplementary Materials

Aptamer Embedded Arch-Cruciform DNA Assemblies on 2-D VS_2 Scaffolds for Sensitive Detection of Breast Cancer Cells

Jinfeng Quan 1†, Yihan Wang 1†, Jialei Zhang 1, Kejing Huang 2, Xuemei Wang 1*, Hui Jiang 1*

[1] State Key Laboratory of Bioelectronics, National Demonstration Center for Experimental Biomedical Engineering Education, School of Biological Science and Medical Engineering, Southeast University, Nanjing 210096, P. R. China
[2] School of Chemistry and Chemical Engineering, Guangxi University for Nationalities, Nanning 530008, China
† These authors are contributed equally to this work.
* Correspondence: E-mail address: xuewang@seu.edu.cn (X. Wang); sungi@seu.edu.cn (H. Jiang).

Signal amplification of AuNPs/VS_2 and VS_2

In order to confirm that AuNPs/VS_2 plays a role of signal amplification in the preparation of the sensor, the effective specific surface areas of GCE and AuNPs/VS_2/GCE are measured by the chronocoulometry (Q-t), and the formula is:

$$Q = 2nFAcD^{1/2}t^{1/2} / \pi^{1/2} + Q_{dl} + Q_{ads} \qquad (1)$$

Where Q is the quantity of electricity, n is the electron transfer number involved in the electrochemical reaction. A (cm^2) and c (mol/dm^3) means the effective area of the electrode and the concentration of the substrate, respectively. D (cm^2/s) represents the diffusion coefficient, and it of potassium ferricyanide is 7.6×10^{-12} cm^2/s. Q_{dl} (C) is a double-layer charge and Q_{ads} (C) is an adsorption charge. According to the calculation in Fig. S1A, the effective specific surface areas of GCE and AuNPs/VS_2/GCE are 0.075 cm^2 and 0.194 cm^2, respectively. It is confirmed that AuNPs/VS_2 greatly improves the effective specific surface area of the electrode, thus providing more active sites to immobilize arch DNA and playing the role of signal amplification.

Two different DPV responses with a target cell concentration of 5000 cells/mL is shown in Fig. S1B. The measurement is carried out in 0.1 mol/L PBS (pH=5.0) containing 1.8 mmol/L H_2O_2 and 2 mmol/L HQ. Curve a represents DPV response of HRP/target cell/cruciform DNA/MCH/arch-DNA structure/AuNPs/GCE. On the basis of curve a, VS_2 is modified on the surface of electrode (curve b). It can be observed that the DPV signal of AuNPs/VS_2 modified GCE is 153.8% of the AuNPs modified GCE, indicating that VS_2 contribute to the signal amplification.

Cytotoxicity and electrophoresis experiments

CCK-8 kit was used to detect the cytotoxicity of DNA assemblies at different concentrations (0 ~ 2 μmol/L) [1]. The toxic effects of arch DNA and cruciform DNA on MCF-7 are detected to be very low, and the cell viability can still be maintained above 90% when the concentration is 2 μmol/L (Fig. S2A).

The assembly of cruciform DNA is validated by natural polyacrylamide gel electrophoresis (PAGE) (Fig. S2B). Lane 1 to 4, lane 5 to 6 and lane 7 to 8 are the results of four single stranded DNA, mixture of two single stranded DNA, and mixture of three single stranded DNA, respectively. When the four single stranded DNA are mixed (lane 9), a band with slower migration than any other mixed DNA are observed, proving the successful preparation of cruciform DNA [2].

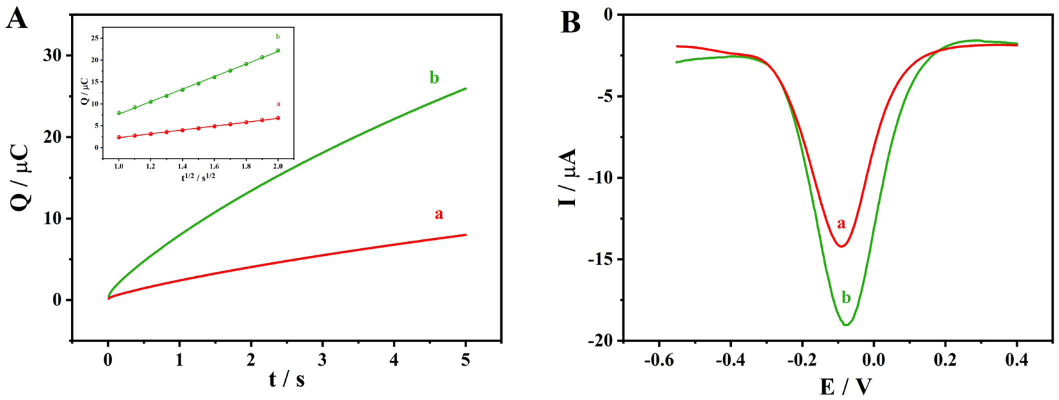

Figure S1 Signal amplification of nanocomposites: (A) Q-t curves of GCE and AuNPs/VS_2/GCE, with Q-$t^{1/2}$ curves of GCE and AuNPs/VS_2/GCE in the inner illustration; (B) The DPV responses of HRP/target cell/cruciform DNA/MCH/arch-DNA structure/AuNPs/GCE (a) and HRP/target cell/cruciform DNA/MCH/arch-DNA structure/AuNPs/VS_2/GCE (b).

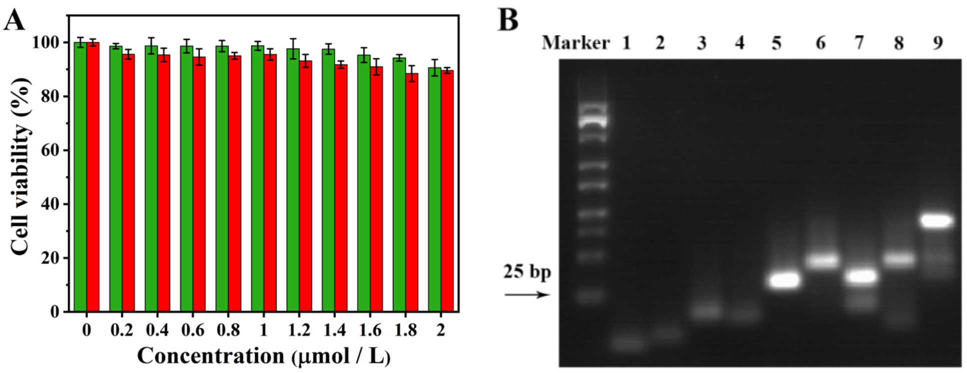

Figure S2 (A) Cytotoxicity test of arch DNA (green) and cruciform DNA (red); (B) Cruciform DNA electrophoresis. Lane 1-9: DNA1, DNA2, DNA3, DNA4, DNA1+DNA2, DNA3+DNA4, DNA1+DNA2+DNA3, DNA2+DNA3+DNA4, DNA1+DNA2+DNA3+DNA4.

Table S1 DNA sequences

Name	Sequence (5′-3′)
DNA1	**biotin**-GGCAAGCTAATGGTGAGCACGGCAGG
DNA2	**biotin**-CCTGCCGTGCTCACCGAATGCTAGGG
DNA3	**biotin**-CCCTAGCATTCGGACTATGGCATGAGTTAGGATCAACTGC
DNA4	CCAGGGTATCCATCTCATGCCATAGTCCATTAGCTTGCC
S1	**SH**-$(CH_2)_6$-TCGGATGACATGCAGTTGATCCTTTGGATACCCTGGTAGGCGATTAAGTA
S2	**SH**-$(CH_2)_6$-TAAGCTTTAGCCTTTACTTAATCGCCTA

Table S2 List of instruments

Instrument	Instrument model	Manufacturer or place of origin
Electrochemical workstation	Autolab PGSTAT302N	Netherlands
Electrophoresis	JY300	Beijing Junyi Oriental Electrophoresis
Gel imaging analyzer	JS-680D	Shanghai Peiqing Technology Co., Ltd
SEM	Zeiss Ultra Plus	Germany
TEM	JEM 2100	Japan
XPS	Thermo ESCALAB 250	USA
XRD	SCXmini	Science of Japan Co., Ltd
Raman Spectroscopy	Renishaw inVia	United Kingdom

SEM: scanning electron microscope; TEM: transmission electron microscope; XPS: X-ray photoelectron spectroscopy; XRD: X-ray diffractometer.

Table S3 Experimental conditions and electrochemical parameters

Method	Electrolyte	Parameters
CV	0.1 mol/L PBS (pH=7.0) including 10 mmol/L $[Fe(CN)_6]^{3-/4-}$ and 0.1 mol/L KCl	voltage range: -0.2 V ~ 0.6 V<br>sweep speed: 100 mV/s
EIS	0.1 mol/L PBS (pH=7.0) including 5 mmol/L $[Fe(CN)_6]^{3-/4}$ and 0.1 mol/L KCl	amplitude: 5 mV<br>frequency: 0.1 Hz ~ 100 kHz<br>voltage: 0.2 V
DPV	0.1 mol/L PBS (pH=5.0) including 1.8 mmol/L H_2O_2 and 2 mmol/L HQ	pulse amplitude: 50 mV<br>pulse width: 50 ms<br>pulse period: 0.2 s
CC	1.0 mol/L KCl including 0.1 mmol/L $[Fe(CN)_6]^{3-}$	time: 5 s
I-t	0.1 mol/L KNO_3 including 0.1% $HAuCl_4$	deposition voltage: -0.2 V<br>deposition time: 25 s

CV: cyclic voltammetry; EIS: electrochemical impedance spectroscopy; DPV: differential pulse voltammetry; CC: chronometric.

References

1. Dong, H. L.; Chen, H. F.; Jiang, J. Q.; Zhang, H.; Cai, C. X.; Shen, Q. M., Highly Sensitive Electrochemical Detection of Tumor Exosomes Based on Aptamer Recognition-Induced Multi-DNA Release and Cyclic Enzymatic Amplification. *Anal Chem* **2018,** *90* (7), 4507-4513.
2. Wang, D.; Chai, Y. Q.; Yuan, Y. L.; Yuan, R., Lattice-Like DNA Tetrahedron Nanostructure as Scaffold to Locate GOx and HRP Enzymes for Highly Efficient Enzyme Cascade Reaction. *ACS Appl Mater Inter* **2020,** *12* (2), 2871-2877.
